# Simultaneous Characterization of Instantaneous Young's Modulus and Specific Membrane Capacitance of Single Cells Using a Microfluidic System

**DOI:** 10.3390/s150202763

**Published:** 2015-01-27

**Authors:** Yang Zhao, Deyong Chen, Yana Luo, Feng Chen, Xiaoting Zhao, Mei Jiang, Wentao Yue, Rong Long, Junbo Wang, Jian Chen

**Affiliations:** 1 State Key Laboratory of Transducer Technology, Institute of Electronics, Chinese Academy of Sciences, Beijing 100190, China; E-Mails: gaochunzy@gmail.com (Y.Z.); dychen@mail.ie.ac.cn (D.C.); luoyana88@126.com (Y.L.); ccmucf@aliyun.com (F.C.); 2 Department of Cellular and Molecular Biology, Beijing Chest Hospital, Capital Medical University, Beijing 101149, China; E-Mails: zhao_xiaoting@126.com (X.Z.); jmcyf627@sina.com (M.J.); yuewentao@gmail.com (W.Y.); 3 Department of Mechanical Engineering, University of Alberta, Edmonton, AB T6G 2G8, Canada

**Keywords:** microfluidics, single-cell analysis, cellular biophysics, instantaneous Young's modulus, specific membrane capacitance

## Abstract

This paper presents a microfluidics-based approach capable of continuously characterizing instantaneous Young's modulus (*E_instantaneous_*) and specific membrane capacitance (*C_specific membrane_*) of suspended single cells. In this method, cells were aspirated through a constriction channel while the cellular entry process into the constriction channel was recorded using a high speed camera and the impedance profiles at two frequencies (1 kHz and 100 kHz) were simultaneously measured by a lock-in amplifier. Numerical simulations were conducted to model cellular entry process into the constriction channel, focusing on two key parameters: instantaneous aspiration length (*L_instantaneous_*) and transitional aspiration length (*L_transitional_*), which was further translated to *E_instantaneous_*. An equivalent distribution circuit model for a cell travelling in the constriction channel was used to determine *C_specific membrane_*. A non-small-cell lung cancer cell line 95C (*n* = 354) was used to evaluate this technique, producing *E_instantaneous_* of 2.96 ± 0.40 kPa and *C_specific membrane_* of 1.59 ± 0.28 μF/cm^2^. As a platform for continuous and simultaneous characterization of cellular *E_instantaneous_* and *C_specific membrane_*, this approach can facilitate a more comprehensive understanding of cellular biophysical properties.

## 1. Introduction

Mechanical properties of the cytoskeleton (*E_instantaneous_* and *E_equilibrium_*) and electrical properties of cell membrane (*C_specific membrane_*) determine the overall cellular biophysical properties [1] and have been correlated with diseases such as malaria and cancer [2,3]. Conventional techniques for cellular mechanical and/or electrical property characterization suffer from limited throughput and cannot quantify mechanical and electrical properties simultaneously [4,5] (e.g., ~20 cells per cell type based on electrorotation [6] and ~10 cells per cell type based on atomic force microscopy [7]).

Advances in microfluidic technologies have enabled mechanical and/or electrical property characterization of single cells in a continuous manner [8–11] (e.g., *E_instantaneous_* values from hundreds of A549 cells [12] and *C_specific membrane_* values from hundreds of H1299 cells [13,14]). However, the majority of reported microfluidic devices were only designed for characterizing either electrical or mechanical properties of a cell, but not both.

Previously, there are three types of devices developed to combine the measurements of cellular mechanical and electrical properties. These approaches were based on principles of microcantilever-based electrodes [15], micropipette aspiration with impedance spectroscopy [16], and constriction channel with impedance spectroscopy [17,18], respectively. Approaches using microcantilever-based electrodes [15] and the combination of micropipette aspiration with impedance spectroscopy [16] have limited throughput and cannot collect data from hundreds of single cells.

In [17,18], single cells were continuously aspirated through a constriction channel (a cross sectional area smaller than cells) while cellular images and single-frequency impedance profiles were obtained simultaneously and translated to cellular mechanical (cellular transition time through the constriction channel) and electrical (impedance amplitude increase during the cellular travelling process within the constriction channel) properties. Although this approach is featured with a higher throughput, which is capable of collecting biophysical data from hundreds of single cells, due to the lack of equivalent mechanical and electrical models, this method cannot interpret raw mechanical and electrical data into intrinsic cellular biophysical parameters (*E_instantaneous_* and *C_specific membrane_*), leading to compromised capabilities in cell status evaluation and cell type classification [19].

**Figure 1.**
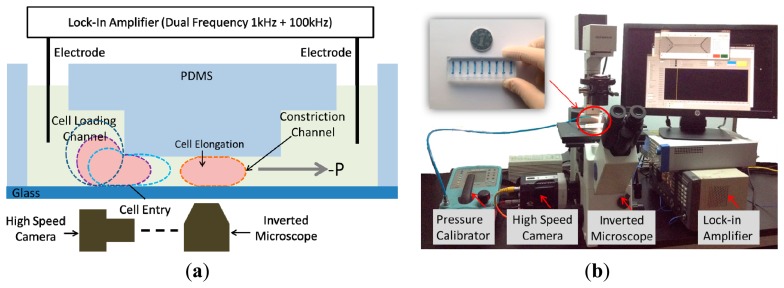
(**a**) Schematic and (**b**) setup of the microfluidic system for continuous characterization of *E_instantaneous_* and *C_specific membrane_* of single cells where cells are aspirated continuously through the constriction channel with cellular entry and traveling images as well as impedance profiles obtained simultaneously and translated to *E_instantaneous_* and *C_specific membrane_* based on proposed equivalent mechanical and electrical models.

Motivated by this challenge, in this paper, we present a constriction channel based microfluidic platform for simultaneous characterization of the intrinsic mechanical and electrical properties of single cells in a continuous manner (see Figure 1). In this platform, single cells were aspirated continuously through a constriction channel while the cellular entry process was imaged by a high speed camera and the two-frequency impedance profiles were measured by an impedance analyzer. Based on equivalent mechanical and electrical models, these raw data were translated to *E_instantaneous_* and *C_specific membrane_*, providing a comprehensive understanding of cellular biophysical properties.

Note that this platform is not a simple integration of previous constriction channel based microfluidic devices capable of characterizing either cellular instantaneous Young's modulus (*E_instantaneous_*) [12] or specific membrane capacitance (*C_specific membrane_*) [13,14]. The simultaneous collection of cellular images and two-frequency impedance data can provide more insights to study cellular behaviors during their entry and traveling processes within the constriction channel and thus provide a more complete characterization of biological cells.

## 2. Materials and Methods

### 2.1. Materials

All cell-culture reagents were purchased from Life Technologies Corporation (Carlsbad, CA, USA) unless otherwise specified. The materials used during device fabrication were SU-8 photoresist (MicroChem Corp., Newton, MA, USA) and 184 silicone elastomer (Dow Corning Corp., Midland, MI, USA). The non-small-cell lung cancer cell line 95C was cultured at 37 °C in 5% CO_2_ in RPMI 1640 medium supplemented with 10% heat-inactivated fetal bovine serum, 100 units/mL penicillin and 100 μg/mL streptomycin.

### 2.2. Device Fabrication and Operation

The microfluidic device consists of a constriction channel (cross-section area: 10 μm × 10 μm) in polydimethylsiloxane (PDMS) elastomer that was replicated from a double-layer SU-8 mold. The detailed fabrication procedures were described in a previous publication [20]. Briefly, SU-8 5 was spin coated and exposed without development to form the constriction channel with a height of 10 μm. Then SU-8 25 (cell loading channel with a height of 25 μm) was spin coated on top of the first SU-8 layer, exposed with alignment and developed, forming the two-layer mold master. PDMS prepolymers and curing agents (10:1 in weight) were mixed, poured on channel masters and baked in an oven for crosslinking. PDMS channels were then peeled from the SU-8 masters, punched to form reservior holes, and bonded to glass slides after plasma treatment.

During operation the device was first filled with culture medium and a droplet of cell suspension (1 × 10^6^ cells/mL) was pipetted onto the entrance of the cell loading channel. A negative pressure of 500 Pa generated from a pressure calibrator (DPI-610 pressure calibrator, Druck, Billerica, MA, USA) was applied to aspirate cells continuously through the constriction channel while silver electrode wires [16] were inserted into the inlet and the outlet of the PDMS device for impedance profile monitoring. Cell images were taken by an inverted microscope (IX71, Olympus Inc., Tokyo, Japan) connected with a Phantom M320S high speed camera (Phantom Inc., Bublin, OH, USA) at 200 frames per second. Two-frequency impedance data (1 kHz + 100 kHz) was sampled by a Model 7270 DSP lock-in amplifier (Signal Recovery, Oak Ridge, TN, USA) with a sampling rate of 20 points per second. Note that impedance data at 1 kHz were used to evaluate the sealing properties of deformed cells with constriction channel walls and impedance data at 100 kHz were used to quantify equivalent cellular membrane capacitance and cytoplasm resistance. All the characterization experiments were conducted within 30 min after cell trypsinization.

### 2.3. Equivalent Mechanical and Electrical Models

Numerical simulations were performed using a finite element package ABAQUS (version 6.11, Dassault Systemes Simulia Corp., Providence, RI, USA) to model cellular entry process into the constriction channel with detailed procedures described in a previous publication [12]. Briefly, the channel walls were modeled as rigid surfaces with a geometrical parameter of *D_channel_* and the cell was modeled as an incompressible solid with a key mechanical parameter of *E_instantaneous_*. The friction on cell-wall interface was modeled with a constant friction coefficient *f_c_*.

**Figure 2.**
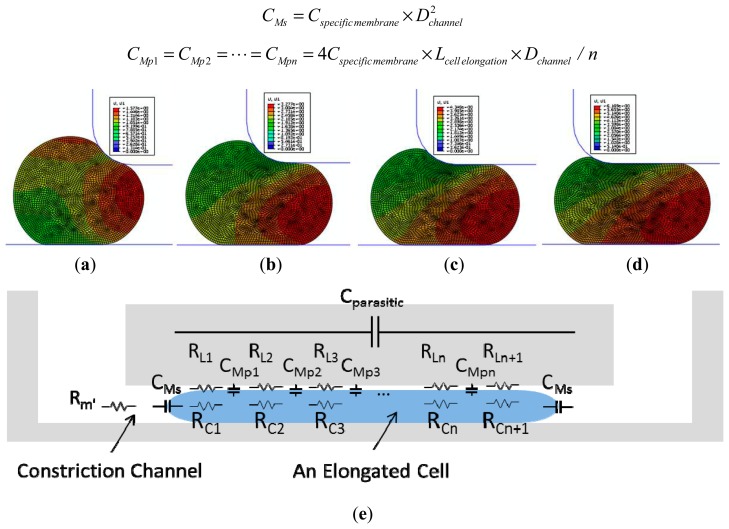
(**a**–**d**) Numerical simulations to model the cellular entry process including (**a**) initial jump into the channel; (**b**,**c**) cellular creep response and (**d**) creep response termination at the transitional position; (**e**) The proposed equivalent circuit model for a travelling cell within the constriction channel where *C_Ms_*, *C_Mp_*_1_, …, *C_Mpn_* and *R_C_*_1_, …, *R_Cn+_*_1_ represent distributed membrane capacitance and cytoplasm resistance with n divisions along the cell elongation direction. Note that *R_L_*_1_, *R_L_*_2_, …, *R_Ln_*, *R_Ln+_*_1_ represent distributed leakage resistors, indicating the sealing properties of deformed cells with constriction channel walls.

Figure 2a–d show the simulation results of the cellular entry process including the stage I of instantaneous jump into the channel (*L_instantaneous_*) (see Figure 2a) and the stage II of cellular creep response (Figure 2b,c), with the ending point at the transitional position (*L_transitional_*) (Figure 2d). Numerical simulation shows that *L_instantaneous_*/*D_channel_* and *L_transitional_*/*D_channel_* are functions of two variables *P_aspiration_*/*E_instantaneous_* (linear function) and ƒ*_c_* as follows (*P_aspiration_* represents aspiration pressure applied to aspirate cells into the constriction channel) [12]:

(1) $$\begin{array}{c} L_{\text{instantaneous}}/D_{\text{channel}}=\left(44.27f_{c}^{2}-37.24f_{c}+13.70\right)\times P_{\text{aspiration}}/E_{\text{instantaneous}}-5.31f_{c}^{2}+2.84f_{c}-0.59\\ L_{\text{transitional}}/D_{\text{channel}}=\left(-60.40f_{c}^{2}+40.05f_{c}-8.68\right)\times P_{\text{aspiration}}/E_{\text{instantaneous}}+1.99f_{c}^{2}+0.03f_{c}+1.60 \end{array}$$

An equivalent circuit model (see Figure 2e) was proposed to model the cellular traveling process within the constriction channel with detailed description shown in a previous publication [13]. Briefly, distributed leakage resistors were represented by *R_L_*_1_, *R_L_*_2_, …, *R_Ln_*, *R_Ln+_*_1_ with n divisions (*n* ≥ 2) along the cell elongation direction while cellular electrical components were represented by *C_Ms_*, *C_Mp_*_1_, …, *C_Mpn_* and *R_C_*_1_, …, *R_Cn+_*_1_, respectively. These distributed equivalent membrane capacitors can be further represented by *C_specific membrane_* as follows (*L_cell elongation_* represents the length of an elongated biological cell travelling within the constriction channel) [13]:

(2) $$\begin{array}{c} C_{\text{Ms}}=C_{\text{specificmembrane}}\times D_{\text{channel}}^{2}\\ C_{Mp1}=C_{Mp2}=\cdots =C_{\text{Mpn}}=4C_{\text{specificmembrane}}\times L_{\text{cell elongation}}\times D_{\text{channel}}/n \end{array}$$

### 2.4. Data Analysis

To measure the elongation length of a cell while it is travelling in the constriction channel, we used a previously developed background subtraction technique to process the images captured by the high-speed camera. This technique consists of a sequence of image processing steps adapted to the context of cell elongation (frame differencing, thresholding, particle removal using erosion, and edge detection along the channel) [21].

Based on the equivalent mechanical model in Section 2.3, raw data of cellular aspiration length were translated to intrinsic cellular mechanical parameter of *E_instantaneous_* and based on the equivalent electrical model in Section 2.3, raw data of two-frequency impedance values and cell elongation length during its travelling inside the constriction channel were translated to intrinsic cellular electrical parameter of *C_specific membrane_*.

## 3. Results and Discussion

**Figure 3.**
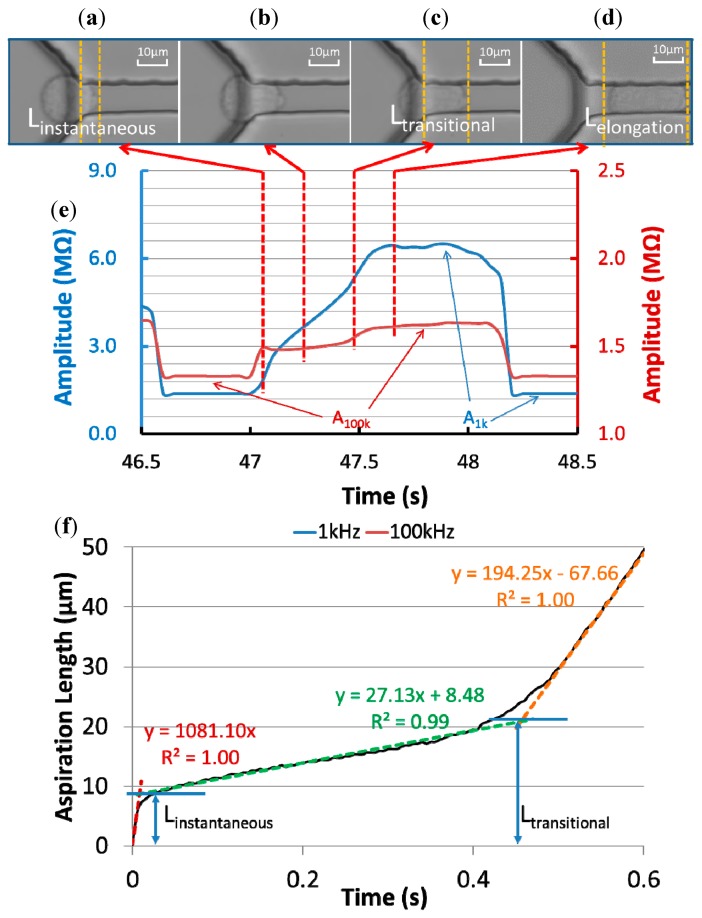
(**a**–**d**) Microscopic pictures of a 95C cell's entry process into the constriction channel with raw impedance data of the same cell shown in (**e**) and aspiration length* vs.* time shown in (**f**). Based on raw data processing, four critical parameters *L_instantaneous_*, *L_transitional_*, *A*_1 kHz_ and *A*_100 kHz_ were obtained.

Figure 3a–d show microscopic pictures of a typical 95C cell's entry process into the constriction channel with raw impedance data of the same cell recorded at 1 kHz and 100 kHz (Figure 3e). Based on the quantified aspiration length (the distance between the leading tip of the cell and the beginning of the constriction channel) *vs.* time (see Figure 3f), the cellular entry process was divided into two stages.

In stage I, an instantaneous jump into the constriction channel was observed (see Figure 3a), characterized by the instantaneous aspiration length (*L_instantaneous_*) in Figure 3f. An instantaneous amplitude jump at 100 kHz and the initiation of a gradual amplitude increase at 1 kHz were also found in this stage. The 100 kHz electric lines were used to penetrate capacitive cellular membrane portions and in stage I its instantaneous increase in amplitude indicates the existence of an equivalent capacitor representing the leading membrane portion, which was sucked into the constriction channel instantly. The 1 kHz electric lines were used to pass around capacitive cellular membrane portions and in stage I, due to the limited aspiration length of the cell under measurement, no significant increase in amplitude at 1 kHz was observed.

After the instantaneous jump, a gradual increase in aspiration length (see Figure 3b,f) was observed due to the cellular creeping behaviour, and this was defined as stage II. During this stage, there is a gradual increase in amplitude at 1 kHz, confirming a gradual increase of the cellular aspiration length. On the other hand, no significant increase in amplitude at 100 kHz was observed, which is due to the fact that the area of the membrane portion sucked into the constriction channel was much smaller than the membrane portion left outside of the constriction channel and thus the contribution of the equivalent capacitor for the portion outside of the constriction channel was negligible.

With further increase in aspiration length while the cell continues to deform due to viscoelastic relaxation, a transitional position quantified as transitional aspiration length (*L_transitional_*) was reached (see Figure 3c), which is the end of the creep stage and the cell rapidly enters the channel. From the perspective of impedance measurement, there is a slope (amplitude/time) increase at 1 kHz, confirming the initiation of accelerated cellular entry into the constriction channel.

In addition, a gradual increase in the impedance amplitude at 100 kHz was noticed for this transition status since as the cell started to enter the constriction channel rapidly, both membrane portions for the leading edge and the trailing edge make contribution to the impedance profiles at 100 kHz.

Figure 3d shows the microscopic picture of the cell travelling within the constriction channel and at this stage, stable impedance amplitudes at 1 kHz and 100 kHz were observed. Furthermore, two critical parameters *A*_1 kHz_ (the amplitude ratio between the stable impedance amplitude at 1 kHz with travelling cells to the basal impedance amplitude at 1 kHz without cells) and *A*_100 kHz_ (the amplitude ratio between the stable impedance amplitude at 100 kHz with travelling cells to the basal impedance amplitude at 100 kHz without cells) were calculated for cellular electrical property quantification.

**Figure 4.**
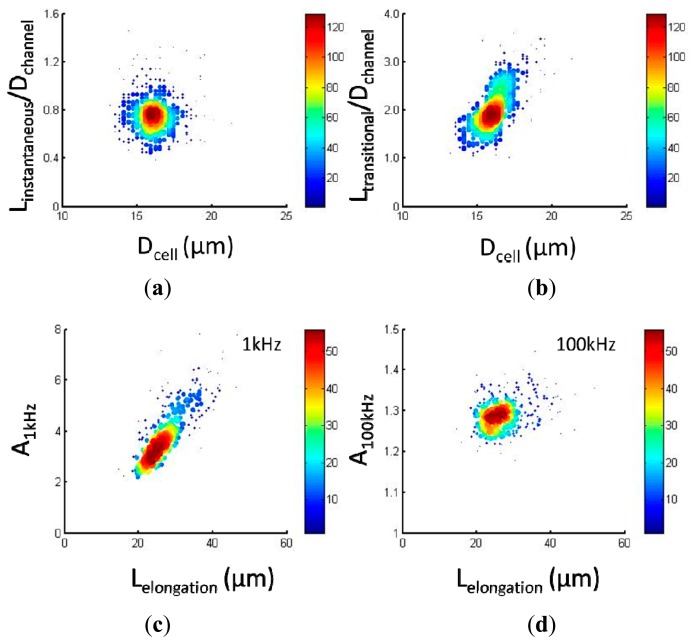
(**a**–**d**) Scatter plots of raw mechanical data of *L_instantaneous_/D_channel_ vs. D_cell_* (**a**) and *L_transitional_/D_channel_ vs. D_cell_* (**b**) as well as raw electrical data of *A_1kHz_ vs. L_cell elongation_* (**c**) and *A_100kHz_ vs. L_cell elongation_* (**d**) for 95C cells (*n* = 354).

Figure 4a,b show scatter plots of cellular mechanical data of *L_instantaneous_*/*D_channel_ vs. D_cell_* (a) and *L_transitional_/D_channel_ vs. D_cell_* (b), respectively, for 95C cells (*n* = 354). Quantified *L_instantaneous_/D_channel_* and *L_transitional_/D_channel_* were 0.76 ± 0.15 and 2.00 ± 0.40 where *D_channel_* represents the characteristic channel dimension (10 μm). It was noticed that with an increase in cell diameter, there is a corresponding increase for *L_transitional_/D_channel_* where *L_instantaneous_/D_channel_* is not dependent on *D_cell_*. This observation was consistent with the previous study where *E_instantaneous_* played a major role in determining *L_instantaneous_/D_channel_* while *L_transitional_/D_channel_* is mainly determined by ƒ_c_ and *D_cell_* [12].

Figure 4c,d show scatter plots of cellular electrical data of *A*_1 kHz_ *vs. L_cell elongation_* (c) and *A*_100 kHz_ *vs. L_cell elongation_* (d), respectively, for 95C cells (*n* = 354). Quantified *A*_1 kHz_ and *A*_100 kHz_ were 3.92 ± 1.09 and 1.29 ± 0.04 where *L_cell elongation_* represents the cellular elongation length during its travelling process within the constriction channel as an indicator of cell sizes. Compared to *A*_1 kHz_, *A*_100 kHz_ was significantly lower since electric lines at 100 kHz effectively penetrate the cellular membrane portions while electric lines at 1 kHz always travel around capacitive cellular membrane portions. With the increase in *L_cell elongation_*, a corresponding increase in *A*_1 kHz_ was observed, further confirming the assumption that electric lines at 1 kHz pass around cells rather than penetrate cellular membrane portions.

**Figure 5.**
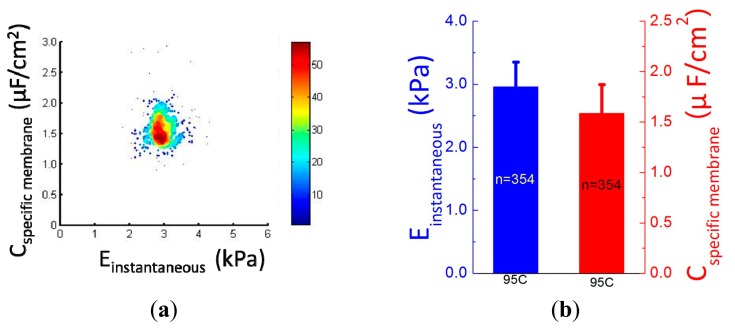

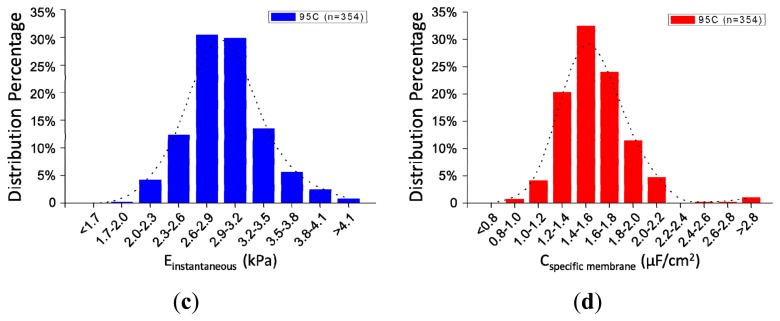
(**a**) A scatter plot of *C_specific membrane_ vs. E_instantaneous_* for 95C cells (*n* = 354) with quantified *E_instantaneous_* of 2.96 ± 0.40 kPa and *C_specific membrane_* of 1.59 ± 0.28 μF/cm^2^ shown in (**b**). (**c**) and (**d**) Distribution percentages of *E_instantaneous_* and *C_specific membrane_* for 95C cells with the peak of *E_instantaneous_* within the range of 2.6–2.9 kPa and *C_specific membrane_* within the range of 1.4–1.6 μF/cm^2^.

Figure 5a shows the scatter plot of *C_specific membrane_ vs. E_instantaneous_*, which were quantified as 2.96 ± 0.40 kPa (*E_instantaneous_*) *vs.* 1.59 ± 0.28 μF/cm^2^ (*C_specific membrane_*) for 95C cells (*n* = 354) (see Figure 5b). Figure 5c,d shows distribution percentages of *E_instantaneous_* and *C_specific membrane_* for 95C cells with the peak of *E_instantaneous_* within the range of 2.6–2.9 kPa and *C_specific membrane_* within the range of 1.4–1.6 μF/cm^2^. These reported values were consistent with previously reported values [12,14], confirming that the proposed microfluidic platform is capable of characterizing cellular intrinsic mechanical and electrical markers simultaneously.

## 4. Conclusions

This paper presented a microfluidic measurement system for characterizing *E_instantaneous_* and *C_specific membrane_* of single cells using constriction channel and impedance spectroscopy. Both microscopic images and impedance profiles confirmed the two-stage cellular entry process into the constriction channel, enabling the quantification of *L_instantaneous_* and *L_transitional_* as key mechanical parameters, which were then translated to *E_instantaneous_*. Furthermore, based on the distributed equivalent circuit model for cellular travelling within the constriction channel, impedance profiles at two frequencies were obtained and translated to *C_specific membrane_*. Compared to previous approaches, this technique is capable of characterizing both *E_instantaneous_* and *C_specific membrane_* in a continuous manner, which can potentially lead to a more complete understanding of cellular biophysical properties.
